# High-level cefiderocol and ceftazidime/avibactam resistance in KPC-producing *Klebsiella pneumoniae* associated with mutations in KPC and the sensor histidine kinase EnvZ

**DOI:** 10.1093/jac/dkaf048

**Published:** 2025-02-19

**Authors:** Jacqueline Findlay, Gabriele Bianco, Matteo Boattini, Patrice Nordmann

**Affiliations:** Medical and Molecular Microbiology, Faculty of Science and Medicine, University of Fribourg, Fribourg, Switzerland; Department of Experimental Medicine, University of Salento, Via Provinciale Monteroni n. 165, Lecce 73100, Italy; Microbiology and Virology Unit, University Hospital Città della Salute e della Scienza di Torino, Turin, Italy; Microbiology and Virology Unit, University Hospital Città della Salute e della Scienza di Torino, Turin, Italy; Department of Public Health and Paediatrics, University of Torino, Turin, Italy; Lisbon Academic Medical Centre, Lisbon, Portugal; Medical and Molecular Microbiology, Faculty of Science and Medicine, University of Fribourg, Fribourg, Switzerland; Swiss National Reference Center for Emerging Antibiotic Resistance (NARA), University of Fribourg, Fribourg, Switzerland

Infections caused by carbapenem-resistant Enterobacterales (CRE), including carbapenemase-producing *Klebsiella pneumoniae*, are increasing globally and constitute a major public health threat.^1^ Within *K. pneumoniae*, KPC-type enzymes are among the most prevalent acquired carbapenemases globally,^1^ and in Italy the ST512 lineage has emerged as the dominant vehicle of KPC dissemination.^2^ The treatment of infections caused by KPC-producing *K. pneumoniae* (KPC-Kp) is particularly challenging due to the fact KPC-Kp often exhibit resistance to multiple classes of antimicrobials in addition to the β-lactams, largely due to their propensity to harbour multiple plasmid-mediated resistance genes as well as the presence of chromosomal mutations in genes that are implicated in resistance (e.g. *gyrA*/*parC*, *ompK35*/*ompK36*). In recent years, the β-lactam/β-lactamase inhibitor combination ceftazidime-avibactam (CAZ-AVI) has been used as a last-resort treatment for KPC-Kp; however, resistance has been frequently reported since its introduction and is usually attributed to mutations within *bla*_KPC_ resulting in enhanced activity against CAZ and/or reduced ability of avibactam to bind.^3,4^ It has also been reported that strains harbouring CAZ-AVI-resistant KPC variants often exhibit reduced susceptibility to the siderophore antibiotic cefiderocol (FDC), likely due to the structural similarities between FDC and CAZ.^5^ Subsequently, treatment of FDC-susceptible KPC-Kp with CAZ-AVI can inadvertently select for both CAZ-AVI and FDC resistance, resulting in both of these last-resort treatments becoming ineffective. Here we describe a case in an Italian hospital where, following treatment with CAZ-AVI, a high-level FDC-resistant CAZ-AVI-resistant KPC-Kp was isolated. This resistance resulted from a combination of mutations within *bla*_KPC_ and the gene encoding the sensor histidine kinase EnvZ.

A 78-year-old man was admitted for an aortic valve replacement and upon routine rectal screening, a CAZ-AVI-susceptible and FDC-susceptible *K. pneumoniae* (KP1) was isolated. The patient was then exposed to meropenem/vancomycin and CAZ-AVI/daptomycin/gentamicin for the treatment of intercurrent infections. After 20 days, a CAZ-AVI-resistant and FDC-resistant KPC-Kp (KP2) was isolated from thoracotomy wound pus.

Susceptibility testing, performed by broth microdilution and interpreted according to EUCAST guidelines,^6^ showed that isolates KP1 and KP2 exhibited CAZ-AVI MICs of 8 and >256 mg/L and FDC MICs of 2 and >256 mg/L, respectively (Table 1). Isolate KP1 was resistant to the carbapenems imipenem (32 mg/L) and meropenem (128 mg/L), whereas KP2 was sensitive to both (0.5 and 1 mg/L). Isolates were subject to Illumina WGS (BioProject No. PRJNA1206977) and subsequent analyses were performed using the Center for Genomic Epidemiology Platform (https://www.genomicepidemiology.org/) as previously described.^7^ Both isolates belonged to ST512, the dominant KPC-Kp lineage in Italy,^2^ and harboured an identical resistance gene content with just one exception: KP1 harboured *bla*_KPC-3_ and KP2 harboured *bla*_KPC-121_. KPC-121 differs from KPC-3 by a serine insertion at position 180, within the KPC omega loop, and this variant has previously been described to confer high-level CAZ-AVI resistance and elevated FDC MICs (Table S1, available as Supplementary data at *JAC* Online).^8^ Analysis of the outer membrane porins OmpK35 and OmpK36 showed that both isolates expressed a WT OmpK35 and an OmpK36 variant with the glycine-aspartate loop 3 insertion that is typical within the ST512 strain lineage.^2^ The KPC-3 and KPC-121 alleles were cloned into the pTOPO vector using primers KPC_F (5′-GATGATGAGCTCTACACCTAGCTCCACCTTCA-3′) and KPC_R (5′-GATGATGGATCCGCAGACTCCTAGCCTAAATG-3′), and transformed into *Escherichia coli* Top10. Subsequent MICs for the recombinant strains showed that the KPC variant was responsible for a 64-fold increase in CAZ-AVI MICs (0.5 to 32 mg/L) but resulted in only a modest 4-fold increase in FDC MICs (0.5 to 2 mg/L). Although KPC-121 did indeed explain the CAZ-AVI MICs and resulted in elevated FDC MICs, it did not explain the high-level FDC MICs observed in KP2, suggesting that another mechanism was contributing. SNP analyses were performed using Snippy (https://github.com/tseemann/snippy) and identified that KP1 and KP2 were indeed closely related, differing by 20 SNPs (Table S2). In addition to the 3 nt insertion converting KPC-3 to KPC-121, a missense mutation was identified within the gene encoding the sensor histidine kinase EnvZ, resulting in a Val147Gly change. The *envZ*/*ompR* two-component system is known to be involved in the regulation of porins in response to medium osmolarity; however, this system has also recently been shown to play a role in iron transport.^9^ Correspondingly, *envZ* has been identified as a hotspot for mutations in *in vitro* FDC mutation studies.^10^ To test whether this mutation was contributing to the high-level FDC MICs the *envZ*/*ompR* genes were amplified from KP1 and cloned into the low-copy vector pACYC184, using primers envZ_ompR_F (5′-gatgatccatgggcacaatttgtagcgcgtaa-3′) and envZ_ompR_R (5′-gatgatccatgggcaacatcctgagcagctaa-3′) before being transformed into KP2. This resulted in significant decreases in the MICs of CAZ-AVI (from >256 to 128 mg/L), and in FDC (from >256 to 64 mg/L), indicating that the *envZ* mutation was indeed having an impact.

**Table 1.** Genotypic and phenotypic characteristics of isolates KP1 and KP2, recombinant strains expressing KPC-3 and KPC-121, and KP2 with complemented WT *envZ/ompR*

		MIC, mg/L															
Isolate	KPC variant	PIP	TIC	AMX	CEF	FOX	CTX	CAZ	CAZ/AVI	FEP	ATM	IPM	MEM	FDC	CST	CIP	GEN
KP1	KPC-3	>256	>256	>256	>256	>256	256	>256	8	256	>256	32	128	2	0.125	128	0.5
KP2	KPC-121	>256	>256	>256	>256	64	64	>256	>256	32	32	0.5	1	>256	0.25	32	0.5
pTOPO-KPC-3	KPC-3	>256	>256	>256	>256	32	256	256	0.5	64	>256	8	16	0.5	≤0.06	≤0.06	0.125
pTOPO-KPC-121	KPC-121	32	128	8	8	2	4	128	32	2	1	≤0.06	≤0.06	2	≤0.06	≤0.06	0.125
KP2/pACYC184	KPC-121	>256	>256	>256	>256	64	64	>256	>256	32	32	0.5	1	>256	0.25	32	0.5
KP2/pACYC184-*envZ*/*ompR*	KPC-121	>256	>256	>256	>256	64	64	>256	128	32	32	0.5	1	64	0.125	64	0.5

AMX, amoxicillin; ATM, aztreonam; CAZ, ceftazidime; CAZ-AVI, ceftazidime-avibactam; CEF, cefalotin; CIP, ciprofloxacin; CST, colistin; CTX, cefotaxime; FDC, cefiderocol; FEP, cefepime; FOX, cefoxitin; GEN, gentamicin; IPM, imipenem; MEM, meropenem; PIP, piperacillin; TIC, ticarcillin.

To investigate any incurred fitness cost caused by the mutations in KP2, competitive growth assays were performed on three biological replicates for each isolate (KP1 and KP2), in iron-depleted Mueller–Hinton media over a 48 h period.^11^ Briefly, broth cultures of KP1 and KP2 were mixed in a 1:1 ratio and grown shaking. Serial dilutions of each culture were grown on LB agar plates both with and without FDC (16 mg/L) and competitive indices (CIs) were calculated as previously described.^11^ The CIs for KP1 and KP2 at 24 and 48 h were 0.019 and 0.006, respectively, indicating that KP2 was outcompeted by KP1. This result could be expected since iron is essential for bacterial cell function, and the limited uptake could be expected to compromise cell fitness.

In conclusion, this study illustrated the isolation of high-level CAZ-AVI and FDC resistance in a KPC-producing ST512 *K. pneumoniae* isolate, following CAZ-AVI therapy. A combination of mutations in the KPC allele and in *envZ* was sufficient to result in a ≥256-fold increase in the FDC MIC although this resistance was shown to come at a significant fitness cost to the bacterium. Despite our findings that isolates KP1 and KP2 were closely related, it is impossible to say with absolute certainty whether KP2 was derived from KP1 or if both isolates were separately introduced to the patient. This report reiterates that a CAZ-AVI-containing treatment may select for FDC resistance and vice versa,^4,5^ and therefore the use of both last-resort molecules should be carefully considered when selecting therapy.

## Supplementary Material

dkaf048_Supplementary_Data

## Supplementary data

Tables S1 and S2 are available as Supplementary data at *JAC* Online.
