# Supplementary material for: High-level cefiderocol and ceftazidime/avibactam resistance in KPC-producing Klebsiella pneumoniae associated with mutations in KPC and the sensor histidine kinase EnvZ
Source: J Antimicrob Chemother. 2025 Feb 19;80(4):1155–7. doi: 10.1093/jac/dkaf048 (PMC11962368; doi:10.1093/jac/dkaf048)
Supplement: dkaf048_Supplementary_Data [file dkaf048_supplementary_data.docx]

|  |  | **Resistance Genes** | |  |
| --- | --- | --- | --- | --- |
| **Isolate** | **ST** | **Beta-lactamase Genes** | **Others** | **Plasmid Replicon Types** |
| KP1 | 512 | *bla*_KPC-3_, *bla*_SHV-11_ | *aac(6’)-Ib*, *aadA2b*, *aph(3’)-Ia*, *fosA6*, *mphA*, *catA1*, *sul1* | FIB(K), FIB(pQIL), FII(K)*, X3, ColRNAI |
| KP2 | 512 | *bla*_KPC-121_, *bla*_SHV-11_ | *aac(6’)-Ib*, *aadA2b*, *aph(3’)-Ia*, *fosA6*, *mphA*, *catA1*, *sul1* | FIB(K), FIB(pQIL), FII(K)*, X3, ColRNAI |

**Table S1**. Genotypic characteristics of isolates KP1 and KP2.

*two copies of the FII(K) replicon were detected.

| **CHROM** | **POS** | **REF** | **ALT** | **GENE** | **AMINO ACID CHANGE** |
| --- | --- | --- | --- | --- | --- |
| contig00001 | 384397 | A | C |  | Asp16Ala |
| contig00002 | 237400 | A | G |  | Val104Val* |
| contig00003 | 15899 | A | T |  | Gln164Leu |
| contig00004 | 197418 | G | C |  | Cys80Ser |
| contig00005 | 110903 | C | A |  | Ala595Glu |
| contig00005 | 112104 | G | C |  | Arg93Gly |
| contig00007 | 168746 | A | G |  | Lys355Glu |
| contig00009 | 49582 | C | A |  | Gly147Val |
| contig00010 | 123107 | A | G |  | Ser270Pro |
| contig00021 | 22288 | G | A |  | Asp262Asn |
| contig00032 | 58594 | C | T |  | Thr2Ile |
| contig00033 | 33609 | CGAT | C |  | Ser182Del |
| **INTERGENIC REGIONS** | | | | | |
| contig00004 | 112557 | A | G |  |  |
| contig00021 | 4524 | C | T |  |  |
| contig00037 | 29151 | A | G |  |  |
| contig00046 | 13530 | CTTC | TTTA |  |  |
| contig00046 | 13586 | CGCCCGGT | AGCTAGGC |  |  |
| contig00046 | 13611 | A | G |  |  |
| contig00076 | 1202 | A | C |  |  |

**Table S2.** SNP analysis of strains KP1 and KP2. Reads from KP1 were mapped against a KP2 assembly using Snippy.

*no amino acid change
